# Feline Coronavirus Antivirals: A Review

**DOI:** 10.3390/pathogens10091150

**Published:** 2021-09-07

**Authors:** Manon Delaplace, Hélène Huet, Adèle Gambino, Sophie Le Poder

**Affiliations:** 1UMR 1161 Virologie, INRAE-ENVA-ANSES, École Nationale Vétérinaire d’Alfort, Maisons-Alfort, 94704 Paris, France; manon.delaplace@vet-alfort.fr (M.D.); helene.huet@vet-alfort.fr (H.H.); a.gambino@anses.fr (A.G.)

**Keywords:** FIPV, feline coronavirus, antivirals, combination therapy, viral escape mutants

## Abstract

Feline coronaviruses (FCoV) are common viral pathogens of cats. They usually induce asymptomatic infections but some FCoV strains, named Feline Infectious Peritonitis Viruses (FIPV) lead to a systematic fatal disease, the feline infectious peritonitis (FIP). While no treatments are approved as of yet, numerous studies have been explored with the hope to develop therapeutic compounds. In recent years, two novel molecules (GS-441524 and GC376) have raised hopes given the encouraging results, but some concerns about the use of these molecules persist, such as the fear of the emergence of viral escape mutants or the difficult tissue distribution of these antivirals in certain affected organs. This review will summarize current findings and leads in the development of antiviral therapy against FCoV both in vitro and in vivo, with the description of their mechanisms of action when known. It highlights the molecules, which could have a broader effect on different coronaviruses. In the context of the SARS-CoV-2 pandemic, the development of antivirals is an urgent need and FIP could be a valuable model to help this research area.

## 1. Introduction

**Figure 1:**
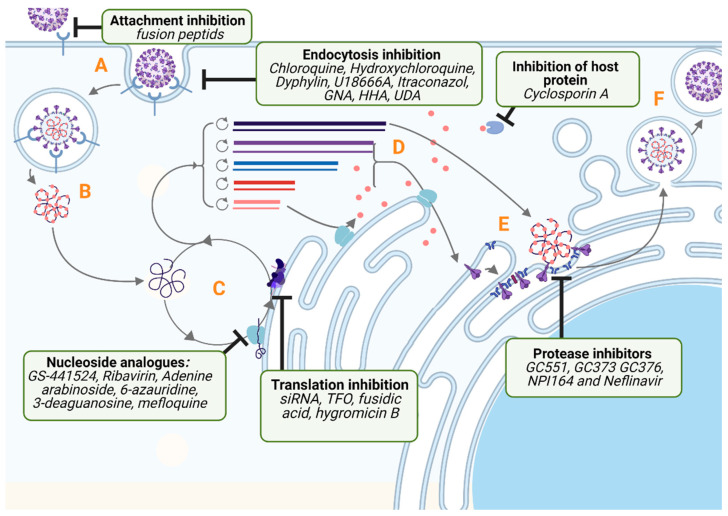
FCoV life cycle and mechanisms of actions of current FIPV antivirals. Letters A–F represent major steps of FIPV replication: (**A**) Attachment to cell and endocytosis. (**B**) Liberation of Ribonucleocapsid following fusion of viral and cell membranes and decapsidation. (**C**) Transcription of viral RNAs and creation of Replication/Transcription Complex. (**D**) Translation of the viral structural proteins. (**E**) Assembly of virion in the endoplasmic reticulum or Golgi apparatus. (**F**) Budding of virus in the ERGIC and exocytosis. HHA: Hippeastrum hybrid agglutinin; UDA: Urtica dioica Lectin; GNA: Galanthus nivalis agglutinin; TFO: Triple Helix Forming Oligonucleotides. Created with Biorender.

Coronaviruses (CoV) are RNA positive single strand viruses belonging to the *Coronaviridae* family within the Nidovirales order. They are classified into four genera: Alpha-, Beta-, Gamma- and Delta-coronavirus genus. Gamma- and Delta-coronaviruses mostly infect birds, with the exception of some mammals. Alpha and Beta-coronaviruses exclusively infect mammals. Coronaviruses display the largest genome among RNA viruses, which ranges from 26 to 32 kB (kilobases). It is a single-strand positive-sense RNA, with a capped 5′ end and a polyA tail on the 3′ end. The genomic organisation is quite similar for all the coronaviruses: the first two-thirds correspond to the ORF1a/b, which encodes the 16 non-structural proteins essential for the virus replication (nsp1–nsp16), followed by the structural genes, always in the same order S (spike)-E (envelope)-M (matrix)-N (nucleocapsid). Interspaced between the structural genes are found additional ORFs encoding for accessory proteins. Those accessory proteins differ in number, size and position on the genome for each viral species of *Coronaviridae*. After binding to the receptor, coronaviruses enter the cell host either via direct fusion at the plasma membrane or endocytosis. Once the nucleocapsid is delivered into the cytoplasm, viral RNA is uncoated for translation of the ORF1a/1b gene into a polyprotein that encodes the 16 non-structural proteins. Synthesized proteins proceed to form the replicase-transcriptase complex (RTC). The RTC will initiate the transcription of full-length negative RNA and negative subgenomic (sg)RNAs. In turn, they will serve as a template for the synthesis of positive full-length RNA and positive sgRNAs. Viral proteins are translated from the positive sgRNAs. After synthesis, they undergo maturation in the Endoplasmic Reticulum (ER) and Endoplasmic Reticulum-Golgi apparatus Intermediary Compartment (ERGIC) and assemble to form the novel viral particles. The viral particles maturation occurs during their transit in the Golgi apparatus and are in fine secreted out of the cell by exocytosis (Figure 1).

Among the six known human coronaviruses, three are highly pathogenic: MERS-CoV, SARS-CoV and SARS-CoV-2. All of them originated from bat coronaviruses [1]. For SARS-CoV and MERS-CoV, intermediate hosts, civet cats and camels, respectively, served as a relay to the human infection. No intermediate host has been determined for SARS-CoV-2 as of yet, but this virus is able to infect multiple animals, including ferrets, minks, non-human primates, dogs, domestic cats and wild *felidae* [2]. As seen with MERS-CoV in camels [3], animals may become a reservoir for the virus and induce recurring transmission to humans. Therefore, it is important to study and find ways to control CoV infection in animals to prevent future epidemics. In addition, animal coronaviruses cause some highly pathogenic diseases in animals, which result in economic losses. Veterinary coronaviruses were the first known CoVs, with the discovery of Infectious Bronchitis Virus in chicken in 1931 [4], 35 years before the report of the first human coronavirus [5], HCoV-229E. Many livestock, wildlife and pet species are affected by different coronaviruses, including bats, cows, horses, camelids, poultry, pigs, dogs and even ferrets [6,7,8,9]. Cats are susceptible to the feline CoVs (FCoV), an Alphacoronavirus [10].

FCoVs are classified into two biotypes based on their pathobiology. Avirulent strains, which usually induce mild or subclinical digestive symptoms, are referred to as feline enteric coronavirus (FECV). FECV is endemic in cats with seropositivity of 20 to 60%, reaching up to 90% of positive cats in multiple cats households [11]. Virulent strains are responsible for feline infectious peritonitis (FIP) disease and are called feline infectious peritonitis viruses (FIPV). FIPV results from spontaneous mutations of FECV, which modify the spike glycoprotein, the 3c accessory protein and potentially the accessory protein 7b. While FECV is largely restricted to the intestinal tract, FIPV is able to infect monocytes and macrophages [12], which leads to the spread of the virus until a systemic infection is established. Two clinical forms of FIP are observed: the effusive form characterized by fluid-filled cavities and the dry form with multiple pyogranulomatous lesions in various organs.

FCoV is also subdivided, based on serological responses, into two serotypes: FCoV-I, the source of most natural infection amongst cats [13], and FCoV-II, resulting from the recombination between FCoV-I and the canine coronavirus, CCoV-II. FCoV-II is usually the laboratory model used due to its ease of replication in cell lines such as CRFK (Crandell-Rees Feline Kidney) or fcwf-4.

Attempts at developing a vaccine have been made, but the efficacy of currently available FCoV vaccines is limited [14,15]. There is still a need for the development of therapeutic compounds against FIPV. Moreover, the SARS-CoV-2 pandemic shows the importance of developing antiviral treatments against coronaviruses. In this perspective, FIP is a good natural animal model to test wide-spectrum antiviral molecules. The purpose of this review is to provide an update on current antiviral molecules tested against FIPV either in vitro or in vivo with a description of their cellular mechanisms of action. Within this framework, we propose to detail the molecules according to the viral cycle step targeted by the antiviral molecules. This review also includes effective immunostimulant molecules. Table 1 lists the values of IC50 (half maximal inhibitory concentration) and CC50 (half cytotoxic concentration) of the different inhibitors addressed in this review.

## 2. FCoV Inhibitors

### 2.1. Inhibition of FCoV Cell Entry

The S protein is responsible for the attachment of CoV to the cellular receptor and the fusion between the cell and the viral membrane for all coronaviruses. The large N-terminal ectodomain of S is subdivided into S1 and S2 subunits that contain the receptor binding domain (RBD) and the fusion domain, respectively. The FCoV-II receptor is the feline aminopeptidase-N (APN) protein, a cell-membrane-bound metalloprotease. The FCoV-I receptor is still unknown. Feline DC-SIGN is also an important entry cofactor in monocytes, at least for feline serotype II strains [39]. Coronaviruses’ entry into cells is complex, as many coronaviruses are able to use both endocytosis and direct membrane fusion to enter host cells [40]. FCoV seems to infect cells via endocytosis in a clathrin- and caveolin-independent but dynamin-dependent manner, at least in monocytes [41]. Molecules inhibiting the virus’s cell entry usually target the interface between the cellular receptor and the viral receptor binding site, harboured by the S protein in CoVs, but some of them inhibit the cellular processes of entry after the viral attachment step.

Different molecules inhibiting the FCoV viral attachment have been attempted. Peptides targeting the S1 domain of Spike, which contains the RBD, were developed. At 160 µM, three peptides corresponding to the amino acids 471–490 (peptide I-S1–9, including the RBD of spike), 541–560 (peptide I-S1–16), and 601–620 (peptide I-S1–22) inhibited around 80% of viral replication of FIPV-I KU-2 strain in fcwf-4 cells. I-S1–9 and I-S1–16 also significantly inhibited FIPV-I UCD-1 and Black strains and, more surprisingly, the FIPV-II 79-1146 strain, despite the sequence heterogeneity between the spike proteins from FCoV serotype I and II. It was therefore hypothesized that the inhibitory effect may be due to a competition between peptides and viral particles to bind to cell receptors, in a non-serotype-specific manner [42].

Peptides corresponding to the Heptad Repeat (HR) region 2 of the S2 subunit of the Spike protein of FIPV-II NTU156 strain were also developed. As HR regions are critical for the fusion of the viral envelope with the host membrane, those peptides could block the virus’s entry. By preincubating FIPV for 1 h with HR 2 peptides, the authors noted a dose-dependent inhibition of infection. Out of five tested peptides, the most efficient, named FP5 had an IC50 of 1.33 µM and a CC50 over 200 µM [16]. None of the peptides were tested in vivo.

Different drugs blocking the endocytosis process of viral particles have also been tested. Among them, diphylin is a natural compound extracted from the plant *Cleistanthus collinus* and acts as a vacuolar ATPase blocker. It has previously been shown to inhibit Dengue virus and Influenza virus [43]. By inhibiting the proton H+ pump in endosomal compartments, diphylin blocks the endosomal acidification and thus the fusion activity of the spike protein, preventing cell infection. In fcwf-4 cells, dyphylin inhibited an FIPV-II strain, NTU156 at 0.6 µM with a CC50 of 6 µM. By delivering diphylin, thanks to polymeric nanoparticles, the antiviral effect and the safety of the molecule increased (IC50 of 9.8 nM and CC50 of 77 µM) [17].

Carbohydrate-binding agents (CBA) attach to N-glycosylated molecules such as the spike and M proteins. Their antiviral activity is due to the inhibition of the viral endocytosis process at the post-attachment stage. CBA compounds such as the plant lectins *Galanthus nivalis* agglutinin (GNA), *Hippeastrum* hybrid agglutinin (HHA) and *Urtica dioica* agglutinin (UDA) have been tested against FCoV-I and II. HHA and UDA inhibited FCoV-I at IC50 of 4 and 2 nM and FCoV-II at 60 and 170 nM, respectively. CC50 was estimated at 24 h 9.9 µM for UDA and >2 µM for HHA. IC50 of *Galanthus nivalis* agglutinin (GNA) was estimated at around 0.012 µM for FCoV-I and 0.2 µM for FCoV-II with a CC50 over 2 µM [18].

Chloroquine (CQ) and its derivative hydroxychloroquine (HCQ) have been described as having an inhibitory effect on early stages infection on different viruses in vitro, including human immunodeficiency virus (HIV), influenza A/H5N1 virus, SARS-CoV and SARS-CoV-2 [44,45,46,47,48]. CQ and HCQ prevent viral replication by blocking the endosome-mediated viral entry. They might also impair post-translational modification of viral glycoproteins, which occur in the endoplasmic reticulum and Trans Golgi Network vesicles. For FCoV, CQ reached maximum efficiency when present before or at the time of infection and started to lose its inhibitory effect when added 1 h post-infection (p.i) of CRFK cells with the FIPV-II 79 1146 strain. Chloroquine had an IC50 of 0.38 µM 24 p.i. while its CC50 reached 82.31 µM after 24 h [22]. Another research team confirmed the antiviral effect of chloroquine on the infection of fcwf-4 and on monocyte cells with the same viral strain. Chloroquine at 10 µM reduced virus titter by 1000-fold in fcwf-4 cells and by 10-fold in macrophages. Hydroxychloroquine, a derivative of chloroquine, is supposed to be less toxic in some animals [49]). In fcwf-4 cells, at 48 h p.i, hydroxychloroquine had a CC50 of 515.7 µM and an IC50 of 48.7 µM against FIPV-I strain KU-2 and of 30.3 µM against FIPV-II. In comparison with CQ, HQ appears to be one-third less cytotoxic [20].

As of yet, in vivo experiments were conducted only with CQ. Three groups of three SPF cats experimentally inoculated with the FIPV-II 79-146 strain were treated with 10 mg/kg of chloroquine (or a saline solution as control), administered subcutaneously. One group was treated 3 days prior to infection and every 3 days thereafter, while another group was treated with chloroquine only 12 days post-infection and every 3 days afterwards. Compared to the control group, both chloroquine-treated groups had slightly lower viral mRNA levels in PBMC 21 days post-infection, as well as slightly lower inflammatory cytokine mRNA levels. Mean numbers of survival days of chloroquine treated cats were higher (34.3 and 31.7 days post-infection) than in the control group (21 days p.i.) though not statistically significant. No treated cats survived the FIPV infection. In addition, despite an improvement of the clinical score of CQ treated cats, an increase of alanine aminotransferase (ALT) due to the treatment was noticed. In conclusion, chloroquine may be an interesting lead to treat FIP but that it may be used in combination with other anti-FIPV drugs, as high doses had severe side effects [19].

### 2.2. Inhibition of the Viral Replication and Transcription

Coronaviruses have a unique system of genome replication and transcription. The first event that follows the release of the genomic RNA is the translation of the ORF1a and 1b. ORF1a is translated into a polyprotein named pp1a and codes for 11 non-structural proteins (nsp1–nsp11asct). The primary translated products are processed by 2 proteases (nsp3 and nsp5) embedded within the polyproteins into 16 nsps. Coronavirus’s nonstructural proteins comprise a helicase and exoribonuclease, which allow the possibility to correct some mutations in their genome, an exclusive characteristic amongst RNA viruses. Together with N and possibly with cellular proteins, the nsps proceed to form the replicase-transcriptase complex (RTC). This structure is tightly connected to the endoplasmic reticulum, as the RTC is made from modified endoplasmic reticulum membranes, induced during Coronavirus infection. The RTC thus has two distinguishable activities: first, as a replicase, it will recognize the genome as a template for copies of genomic strands, and second, as a transcriptase, it will generate copies into subgenomic mRNAs for viral translation. Coronavirus viral polymerase synthesises both genome-length strand and multiple subgenomic RNAs (sgRNAs) through a unique discontinuous transcription mechanism. The sgRNAs serve as templates for the translation of both structural and accessory proteins [50].

Antivirals targeting viral replication follow different strategies. The inhibition the viral polymerase is a classic mechanism of action used for antiviral therapies against HIV or HCV.

#### 2.2.1. Nucleoside Analogues

Nucleoside analogues are similar to natural nucleosides, but the differences with a standard nucleoside induce different base pairing when used to synthesize DNA/RNA and inhibit further viral replication. They are often used as antivirals (HIV, Herpesvirus, etc.). Cells are able to remove and correct the presence of these analogues, whereas viruses cannot.

##### GS-441524

GS-441524 is an adenosine analogue and the active form of Remdesivir (Gilead). GS-441524 has been tested in vitro and in vivo for its effect against FIPV. In vitro, GS-441524 was able to completely inhibit RNA expression of FIPV-II 79 1146 in CRFK cells at 10 µM and partially at 1 µM. No cytotoxic effect of the compound was noticed up to 10 μM. This compound was efficient when added 1 h post-infection. In macrophages obtained from ascites fluid of naturally infected cats, 10 µM of GS-441524 reduced viral RNA by 1000-fold 72 h after treatment.

In 10 cats experimentally infected and expressing clinical symptoms of FIP, a daily treatment during 2 weeks of 2 mg/k or 5 mg/kg of GS-441524 2 to 3 days after symptoms apparition resulted in a remission of FIP symptoms. A recurrence of clinical signs occurred for two cats, four or six weeks after stopping the treatment. With further administration over two weeks, these cats also recovered from their symptoms. All cats remained asymptomatic 10 months after treatment [25].

A clinical study on 31 cats suffering naturally from various forms of FIP (nervous form excluded) was conducted. Five cats died or were euthanized due to severe symptoms. After 12 weeks of treatment at 2.0 mg/kg per 24 h, 18 cats remained healthy 44 weeks later. Eight cats relapsed within 3 to 84 days and were treated again for another 2 weeks at 2 mg/kg or 4 mg/kg. Two of them relapsed a second time and were treated a third time at 4 mg/kg. All but one remained healthy afterwards [23]. The monitoring of viral RNA demonstrated a rapid decrease of the viral amount within one week of treatment in most cats. The serotype of FCoV was not identified, but as serotype-I is the most prevalent in natural infections, cats included in this study were probably infected by FCoV-I strains. A second clinical study was conducted a year later on four cats presenting a neurological form of FIP. The cats were treated with 5–10 mg/kg of GS-441524 for at least 12 weeks with daily injection. Three of the four cats treated were alive 254 to 528 days after treatment. The last cat had to be euthanized after relapses following two sessions of treatment, 216 days after treatment initiation [24]. GS-441524 may also be efficacious to treat nervous forms of FIP but with a higher dose up to 10 mg/kg than for the non-neurological forms.

Even though FIP diagnosis was not confirmed in all cases included in the two field studies conducted, GS-441524 appears as the most encouraging FIPV inhibitor with successful treatment on different FIP forms in natural infections. Despite these promising results, GS-441524 has not yet been authorized for veterinary treatment.

Another retrospective study on cats highly suspected of FIP showed that 23 out of 24 cats treated with GS-441524 recovered from FIP. The remaining cat relapsed after 4 weeks of treatment, and its owner decided to not pursue further treatment [51].

Another GS-441524-like drug named Mutian X has also been administered in multiple-cat households, with the aim to stop fecal shedding FCoV and prevent viral transmission in catteries. An observational study followed FCoV fecal secretion in 29 cats treated orally with the Mutian X in catteries [52]. A dose of 4 mg/kg per 24 h significantly reduced the excretion of FCoV within four days. This could be an interesting alternative to avoid viral transmission among cats and in turn prevent the development of FIP. Mutian X was also used to treat one cat with success. A 2-year-old male cat presenting a uveitis was diagnosed with a non-effusive FIP. He was treated with systemic and topical prednisolone for 6 days, and with 8 mg/kg of Mutian X per 24 h given orally for 19 days (days 6 to 25), then 6 mg/kg for 21 more days. Fifty days after the beginning of Mutian X treatment, symmetric dimethylarginine (SMDA), an early indicator of kidney disease, peaked at 28 µg/dL, twice the reference range (<14 µg/dL). Since the cat’s uveitis and other symptoms (weight loss, anemia, elevated alpha-1 acid glycoprotein level) had resolved, the clinicians decided to stop Mutian X treatment and replaced it with 100,000 units of feline interferon omega (IFNω), given orally. SMDA fell to 19 µg/dL 35 days after the discontinuation of Mutian X treatment, which could indicate that elevated SMDA level is a side effect of Mutian treatment. IFNω treatment continued for at least 100 days at the time of the article writing. Three out of four cats living in the same household as the FIP cat were found to be shedding FCoV and were preventively treated with 4 mg/kg of Mutian for five days, which successfully stopped shedding in feces. All six cats were alive and well 6 months after diagnosis and treatment initiation [53].

Given the potential success of GS-441524 in treating FIP cats, many cat owners have independently treated their pets with unlicensed GS-441524-like drugs. A recent study conducted a survey via the Internet of owners who have attempted to treat FIP with unapproved GS-44-1524 drugs. According to their reports, 54% of them considered their cats to be cured of FIP, and most of the treated animals were alive during the 12 weeks of monitoring. This study highlights the potential benefit of GS-441524 drugs, but many concerns remain about the widespread use of these unapproved treatments, including the potential emergence of resistant viral strains [54].

##### Other Nucleoside Analogues

Mefloquine is another adenosine analogue used as a treatment and prophylactic anti-malarial drug for people. Its antiviral mechanism of action is not fully understood. In vitro, its efficiency was maximal when added prior to or concurrent with infection, but remained greater than 50% when added at the latest tested time point of 6 h post-infection with the FCoV-II 79-1683 strain in CRFK cells. Its IC50 was 0.74 µM at 24 h p.i and 5.71 µM 48 p.i with a CC50 of 15.13 µM at 24 h [22]. Given these encouraging results, the pharmacokinetics of mefloquine was recently investigated. Seven healthy cats were orally administered at 62.5 mg four times over the course of two weeks. The peak plasma concentration was 2.71 µg/mL 15 h after a single oral dose. When administered with food, the peak means climbed to 4.06 µg/mL, which could be sufficient to inhibit FIPV. Cats treated with mefloquine showed a mild increase of serum symmetric dimethylarginine. Some cats given mefloquine without food have vomited [50]. Given the good safety data, clinical trials on cats suffering from FIP could be engaged.

Ribavirin, a guanosine analogue, is widely used in human antiviral therapy. It efficiently reduced FIPV 79-1146 strain infection in vitro with a maximal effect of over 10,000-fold of FIPV titer reduction when added 1 h post-infection. The necessary dose, 50–100 mg/mL, was, however, cytotoxic [51]. In vivo, 50 kittens were experimentally infected with the FPV 79-1146 strain and treated 18 h post-infection with 16.5 mg/kg given per os, intramuscularly or intravenously, once daily for three consecutive days, then once each week for three weeks. Cats treated with ribavirin demonstrated exacerbated symptoms. The toxicity and severe side effects induced by ribavirin may be responsible for these observations [55]. Ribavirin is not recommended for FIP treatment.

Other nucleoside analogues were also tested in vitro on the FIPV 79-1146 strain: the adenine arabinoside purine analogue, used in treatment against HHV-1 and HHV-3 [56] and the 6-azauridine, an analogue of uridine and 3-deaguanosine, an analogue of guanosine. Adenine arabinoside inhibited significantly FIPV in vitro with an IC50 of 125 µg/mL at 72 h p.i. but only when delivered before or at the same time as the viral inoculum. No cytotoxic effect was observed up to 250 µg/mL [28] 3-deazaguanosine, and 6-azauridine inhibited FIPV in vitro with respective IC50 of 10.37 µg/mL and of 0,16 μg/mL. Their cytotoxicity ranged from 323.32 for 3-deazaguanoside to 19.4 µg/mL for 6-azauridine. The antiviral effect of these nucleoside analogues was lost if added 1 h after viral infection [29].

#### 2.2.2. siRNA

siRNA is an antiviral therapeutic concept based on RNA interference. It consists of the degradation of mRNA via sequence-specific complementary short RNA. As such, siRNA can be extremely selective and specific to even novel viruses. However, RNA viruses with their high mutation rate and low sequence fidelity rapidly become resistant to siRNA. Success against RNA viruses has been observed by combining multiple siRNAs against different targets. Some are even being tested in clinical trials against HIV, HBV or HCV [57], for example.

A combination of 30 nM of a siRNA mix against the 5′ leader sequence, nucleocapsid and membrane RNA sequences inhibited infection by over 100-fold in the pre-treatment of CRFK cells infected with FIPV-II 79-1146. At 24 h p.i, 5 nM of each siRNA against the nucleocapsid or untranslated 5′ region were sufficient to inhibit over 80% of FIPV infection [30]. No viral escape was notified after five passages in cells. Single or dual siRNA therapy was less efficient and rapidly led to the emergence of viral escape mutants [58]. No in vivo experiments were attempted.

Triple Helix Forming Oligonucleotides (TFO) are homopyrimidine that bind in the major groove of duplex nucleotides strands on specific targets, typically 10–30 nt in length with purine on one strand and pyrimidine on the other [59]. Although TFOs are usually complex with double strand DNA, they can also bind to double strand RNA or DNA–RNA hybrids. Five TFOs targeting different regions of the FIPV genome (based on the strain FIPV 79-1146) were tested in vitro. Three targeted ORF1a/b, one the 3′UTR sequence and the other the 5′UTR sequence of the genome. CRFK cells were transfected with 100 nM of TFOs 6 h before infection. All TFOs inhibited FIPV significantly by approximatively 10^5^-fold except for the one directed against the 3′UTR sequence. The authors tested the TFO1, targeting the 5′UTR, at different concentrations. It was observed that 25 nM of TFO1 did not significantly reduce FIPV infection, while 50 nM reduced infection by 10^5^-fold [60]. The antiviral effect of TFOs seems to be more pronounced than with siRNA treatment, however, further studies are needed, especially to investigate the possibility of viral escape emergence.

### 2.3. Protein Synthesis Inhibitors

Two proteins synthesis inhibitors have been tested in vitro: fusidic acid and hygromycin B. Both are antibiotics. The fusidic acid binds to the elongation factor G (EF-G) and prevents its replacement in the ribosome, thereby inhibiting protein synthesis. Hygromycin B is an aminoglycoside inhibiting protein synthesis by stabilizing tRNA in the ribosomal acceptor site. Both molecules reduced FIPV-induced ECP by three- to sixfold in vitro when added at 2.6 µg/mL for Hygromycin B and 50 µg/mL for fusidic acid. Both molecules had a similar effect when added 1 h before, during or 1 h after infection [29].

### 2.4. Protease Inhibitors

The post-translational maturation of viral non-structural proteins is carried out by two main viral proteases. Nsp3 allows the release of nsp1, nsp2 and nsp3. The second protease is nsp5, which is responsible for the release of the remaining 13 nsps. Due to its similarities with the 3C proteinase of the Picornavirus, nsp5 is often designated as 3C-like proteinase (3CLpro).

Different derivatives of peptidyl compounds targeting 3CL pro could inhibit FIPV in vitro when adding just before the infection. IC50 of compounds ranged from 0.02 µM to 0.86 µM with a CC50 at 24 h ranging from 20 µM to more than 150 µM [61]. Among these compounds, the most promising were GC376 and GC373. With a respective IC50 of 0.07 and 0.15 µM against FIPV-II at 24 h p.i and a CC50 superior to 500 µM, their therapeutic index (calculated from the ratio of IC50/CC50) was excellent. Their antiviral activity increased when the molecules are added in pretreatment [33].

GC376 and NPI64, a homologous compound to GC376 with an additional residue of 1-naththylalanine, have been studied in vivo. GC376 inhibits 3CLPro by forming a covalent bond with a cysteine residue of the protease, after conversion of GC376 in an aldehyde form [62].

Before in vivo studies of FIP treatment, the pharmacokinetics of GC376 and NPI64 in two SPF cats have been studied. A peak plasma level of GC376 was observed two hours after subcutaneous injection of 10 mg/kg, and drug concentrations remained above IC50 (8 ng/mL) for 18 h. NPI64 was less readily absorbed into the blood, and the plasma drug level remained above IC50 (12 ng/mL) only for 12 h post-injection of 5 mg/kg. SPF cats injected twice a day with 10 mg/kg for four weeks with GC376 showed no adverse effects. Given these results, efficacy studies were conducted with GC376. In total, eight SPF cats were experimentally infected with FIPV-I. In the first group of four cats, treatment with 5–10 mg/kg of GC376 twice a day started when moderate FIP symptoms appeared (starting at day 15 p.i for the first cat and day 20 p.i for the last one). In the second group, treatment started when severe FIP symptoms appeared, notably, the occurrence of ascites fluid. These cats were first treated with palliative care (the anti-inflammatory compound meloxicam as well as subcutaneous fluids), then, starting at day 18 for the first cat and day 21 for the last, palliative treatments were replaced by twice-daily injection of GC376 at 5–10 mg/kg. All cats were treated until day 34 p.i., except for two cats from the second group that had to be euthanized due to severe symptoms. In all six remaining cats, symptoms disappeared after treatment, and no relapse was observed up to 8 months after the end of treatment. The two euthanized cats showed signs of pancreatitis after necropsy but mild or no lesions typical of FIP. Observed pancreatitis could be a complication due to the meloxicam treatment [60].

Given these encouraging results, GC376 was evaluated on client-owned cats suffering from FIP. FIP diagnosis was based on clinical symptoms, hematological and biochemical abnormalities and detection of the virus by RT-qPCR. Cats with neurological symptoms were excluded. Cats were treated with 15 mg/kg of GC376 injected subcutaneously twice-daily. Out of the 20 treated cats, symptoms improved for 19 of them within 2 weeks of treatment. Nevertheless, 1 to 7 weeks after treatments, symptoms recurred, and the cats were treated for a minimum of 12 weeks. Thirteen cats relapsed and failed to respond to treatment within one to seven weeks of initial or repeat treatment. Eight of those thirteen cats developed severe neurological FIP, and five had recurrence of abdominal lesions. The FIP diagnosis was confirmed at necropsy for 12 of them. Five cats (aged 3.3–4.4 months) with wet FIP were still in remission 5–14 months after the 12 weeks treatment. Another kitten had a relapse 10 weeks after initial treatment but was responding to repeated treatment at the time of writing of that study. Another 6.8-year-old cat relapsed three times and required over 10 months of treatment before remission. In total, 7 out of the 20 cats studied survived after treatments with GC376, mostly kittens with the wet form of FIP [61]. Analyses of viral protease sequences in five treated cats did not demonstrate any mutations except for one. Three mutations were observed in the viral protease sequence after a long duration of treatment, but only one seems to slightly diminish the efficacy of GC376 [63]. Despite these reassuring results regarding the low emergence of resistance variants, surveillance of such events remained to be carried out when the long-term antiviral treatments were implemented.

Another protease inhibitor, nelfinavir, a compound used to treat HIV-infected patients, has been shown to efficiently inhibit SARS-CoV. In vitro, nelfinavir inhibited FCoV-II NTU156 in fcwf-4 cells with an IC50 of 8.19 µM. This was higher than the inhibition of SARS-CoV (IC50: 0.048 µM), probably due to the sequence differences between FCoV and SARS-CoV 3CLPro protease. Indeed, only 7 out of 18 binding sites for nelfinavir identified on SARS-CoV 3CLPro are conserved in FCoV. In contrast to what was observed with SARS-CoV [64], nelfinavir lost its antiviral activity against FCoV when added after virus entry into cells. Encouragingly, nelfinavir was also given orally to nine healthy FCoV shedding cats at 6.25 mg to 50 mg/kg/day without any side effects. The authors did not specify the impact of nelfinavir on FCoV shedding [35].

### 2.5. Inhibition of Host Cellular Proteins

Designing antiviral directly targeting RNA viruses is challenging due to their high mutation rate and inhibition escape potential. A possible strategy to avoid this problem is to design compounds targeting the host proteins that are used by the virus. As such, viral escape through mutation is less likely.

U18666A is a cationic amphilic drug that suppresses cholesterol transporters and prevents cholesterol release from lysosomes by targeting the host Niemann-Pick C1 (NPC1) protein, which is involved in cellular cholesterol trafficking. The CC50 value of U18666A was 97.6 μM. U18666A had an inhibitory effect on four FCoV-I strains in pre-treatment at doses ranging between 0.2 and 20 μM but no effect on FcoV-II replication in vitro [65]. U18666A was later tested in vivo in SPF cats. After infection with FIPV-I KU-2 strain, U18666A was injected subcutaneously at 2.5 mg/kg on day 0, and 1.25 mg/kg on days 2 and 4. PBS was instead injected to the control group. Two out of five control cats developed FIP, as did one out of five cats in the treated group. All cats regardless of treatment shed FIPV RNA in their feces. This in vivo experience was declared non-conclusive due to the low number of symptomatic cats in the control group [36].

Itraconazol (ICZ), such as U18666A, interacts with cellular NCP1 (Niemann-Pick C1 protein) and induces cholesterol accumulation in lysosomes, inhibiting host cellular cholesterol transport. Alternatively, itraconazol inhibits ergosterol biosynthesis by acting on the oxysterol-binding protein (OSBP) [66]. Itraconazol is an antifungal compound used in veterinary medicine to treat fungal infection in cats and dogs. ICZ inhibited FCoV-I replication efficiently (with a selectivity index between 349 and 1426 for three FCoV-I strains) both as a pre-treatment of cells and when added 1 or 3 h post-infection. However, FCoV-II replication was only slightly decreased in the presence of ICZ [37]. As an approved veterinary treatment, the pharmacokinetics of ICZ in cats has previously been described: 10 mg/kg per os was associated with a blood peak concentration of 1.6 µM of ICZ, which reached 4.8 µM when cats were treated twice in a 12 h span [67]. As the IC50 necessary to inhibit FCoV-I was below 1 µM, ICZ could be an interesting therapeutic agent. A study evaluated the antiviral effect of itraconazol in combination with ADA, an anti-human TNFα (Tumor Necrosis Factor). Three out of ten SPF cats infected with FCoV-I KU-2 strain developed FIP symptoms. Treatment with ADA and ICZ started at the onset of symptoms, which corresponded to days 21, 32 and 34 post-infection for the three cats. Each cat was treated twice in the first 4 days with 10 mg of ADA and daily with 50 mg of ICZ during each of the 30 days of the treatment. During this time, clinical symptoms improved for two cats while the third cat, the only one with ascites, did not respond to the treatment and had to be euthanized [38]. In a case report, a client-owned kitten with effusive FIP was treated with 10 mg/kg of ICZ twice a day and 1 mg/kg of prednisolone once a day. Fecal FCoV RNA and pleural fluid accumulation initially decreased but the cat start showing neurological symptoms and had to be euthanized after 38 days [68]. A post-mortem examination confirmed the FIP diagnosis. Despite some improvement of the symptoms, itraconazol failed to cure FIP disease.

Cyclosporine A (CsA) is an immunosuppressive macromolecule that binds to cyclophilins, chaperone proteins able to catalyze the cis/trans conformation change of proline residues. This interaction blocks the calcineurin pathway, which in turn inhibits the translocation of nuclear factor of activated T cells from the cytosol into the nucleus. The antiviral effect of CsA is probably due to its interaction with cellular cyclophilins, as numerous studies demonstrated they are essential factors for coronaviruses’ lifecycles. In fwcf-4 cells, CsA was cytotoxic at a high dose (CC50 of 14.1 µM) and inhibited FIPV in a dose-dependent manner at CsA concentrations between 0.16 and 10μM in post-infection treatment [69]. CsA treatment was attempted in a client-owned cat suffering from an effusive form of FIP. Administration of CsA ranged at doses from 25 mg/kg to 75 mg/kg. The symptoms and the effusion regressed, but relapse occurred at day 251 post-treatment, and the cat died of respiratory failure after 264 days of treatment [70]. However, without post-mortem examination, the diagnosis of FIP was not confirmed.

### 2.6. Immunomostimulant Molecules

Immunomodulation is a strategy used for many antiviral therapies, including veterinary medicine. Interferons (IFN) are widely used in this context. A feline interferon omega (fIFNω) is even commercially available. In vitro, fIFNω reduced the viral yield less than 10-fold when cells are treated continually for 48 h p.i. with 10,000 U of IFN molecule. The antiviral effect was not observed when the molecule was used in pre-treatment [71]. In vivo, a placebo-controlled study was conducted on 37 diagnosed client-owned FIP cats [72]. Cats from the interferon group (21 cats) received 106 U/kg (0.1 mL/kg) flIFNω subcutaneously every 24 h for a week, then the same dose once a week until euthanasia. The control group received a saline solution. All cats were treated with glucocorticoïds. No benefit of the treatment was observed. Human Interferon-alpha (hIFNα) had an inhibitory effect in a dose-dependent manner in vitro [73], but no inhibitory effect was observed in an experimental trial [74]. In conclusion, the main interest of IFN could be in combination with other antiviral molecules (see below) [75].

Polyprenyl Immunostimulant (PI) is a compound used in the treatment of feline herpesvirus. Its antiviral effect may be due to the upregulation of Th-1 cytokines. In a field study, 60 cats diagnosed with a non-effusive form of FIP were treated with PI at 3 mg/kg orally three times per week. While no cats recovered, seven cats survived for over 300 days, with a record of 1829 days for one cat. Importantly, cats treated concomitantly with PI and corticosteroids survived a mean of 47.5 days, while cats treated with PI and without corticosteroids survived a mean of 201 [76]. There may be an adverse effect of treating cats with corticosteroids and PI simultaneously.

### 2.7. Combination Therapy

Another solution to the escape potential of viruses against therapeutics antivirals is combination therapy. Efficient treatments for chronic infection with HCV [77,78] and HIV [79] are based on this strategy. A combination of compounds targeting different steps of the viral replication cycle can have synergistic effects and allow for the use of a lower dose than monotherapy, with potentially lower cytoxicity or side effects.

This was the case for the combination therapy between nelfinavir and GNA for example. When added to cells pre-infected with an MOI 0.1, nelfinavir and GNA had no significant antiviral activities below cytotoxic doses. However the addition of Neflinavir at 9.41 μM with doses of GNA ranging between 0.12 and 0.48 nM, or the addition of 0.48 nM of GNA with Nelfinavir doses between 6.27 and 9.41 μM completely inhibited FCoV infection, without cytotoxicity [35].

A yet-to-be-peer-reviewed study [80] screened 89 compounds against FIPV-II (including some described in this review) and then evaluated the antiviral activity with different combinations of compounds. GC376 at 20 µM was used in combination with nine other compounds at 10 µM. The combination with the most efficient antiviral activity was GC376 at 20 µM with 10 µM of amodiaquine, a molecule used in malaria treatment. This combination reduced FIPV RNA by 10^7^-fold and showed a great synergetic effect compared to monotherapy.

Takano et al. evaluated the antiviral effect of a combination of HCQ and Interferon omega (IFN-ω) on fcwf-4 cells. After 24 h exposure to 100 µM of hydroxychloroquine and 10,000 U/mL of IFN-ω, cell viability was 95%. The antiviral effect of this combination was then evaluated on three FIPV-I strains, UCD1, UCD4 and KU2, as well as on FIPV-II 79-1146. HCQ at 100 µM and IFN-ω at 10,000 U/mL incubated independently for 1 h reduced viral titers by approximatively 10-fold in each case, but for the 3 tested FIPV-I and FIPV-II strains, a combination of HCQ and IFN-ω strongly reduced viral replication by more than 1000-fold, at least [20].

Lee et al. also observed a synergic inhibitory effect when cells infected with FIPV-II were treated with a combination of the peptide FFP5 targeting the HR2 domain of the spike protein and Interferon-alpha (IFN-α). The combination of 10 μM FP5 with 1000 IU of IFN-α with reduced the viral titre up to 10^4^-fold [16].

Despite these promising results, combination therapies were not attempted, to date, in vivo in FIP infected cats.

## 3. Conclusions

As many authors noticed, one of the challenges in the search for an effective therapy is that for many compounds, FIPV-inhibitory doses can be toxic or have severe side effects. Since no approved treatments exist as of yet, efforts in finding adequate treatment for FIP have to be continued. As has been suggested, a combination of antiviral molecules with overall lower doses could be an interesting lead. Another advantage of combination therapy would be to avoid the emergence of antiviral escape mutants, which could be a real concern of the use of some molecules based on targeting viral proteins or nucleoside analogues.

Outside the obvious interest for cats and cat owners, developing antivirals against FIPV has proved to be a useful proof of concept for the development of antivirals against human coronaviruses. Table 2 lists the inhibitors with a broad spectrum on different coronaviruses. For example, a recent study described the antiviral effect on SARS-CoV-2 of an improved version of GC376, a compound initially tested against FIPV [81]. GS-441524 is a parent nucleoside to Remdesivir that has been considered for the SARS-CoV-2 treatment [82]. Some of these inhibitors, such as lectins, have the potential to inhibit all coronaviruses from the different genera [83]. However, most studies have been conducted in vitro and in vivo considerations in animal models are necessary to avoid disappointment such as the hydroxychloroquine treatment against SARS-CoV-2.

The SARS-CoV-2 pandemic, which follows on the of SARS-CoV and MERS-CoV epidemics, has highlighted the need to prepare for the emergence of novel coronaviruses in the human population. Developing antivirals to treat FIP and other veterinary coronaviruses will not only save the lives of many pets but will also help to prevent future coronavirus outbreaks.

## Figures and Tables

**Table 1:** Compounds with antiviral effect against FCoV.

Compound	Strain	IC50 ^1^ (µM)	CC50 ^1^ (µM)	SI	In Vivo Test	Ref
HR peptides (FP5)	FIPV-II NTU156	1.33	>200	>150		[16]
Dyphillin	FIPV-II NTU156	0.59	5.99	10.15		[17]
Dyphillin nanoparticle	FIPV-II NTU156	0.098	77.26	7883.67		
GNA	FIPV-II 79-1146	0.2 °°	>2 **			[18]
	FIPV-I Black	0.012 °°				
HHA	FIPV-II 79-1146	0.06 °°	>2 **			
	FIPV-I Black	0.004 °°				
UDA	FIPV-II 79-1146	0.17 °°	2.2 **			
	FIPV-I Black	0.02 °°				
Chloroquine	FIPV-II 79-1146	21.2 *	325.3 (timing not specified)	15.34	SPF cats [19]	[20]
Chloroquine (diphosphate)	FIPV-II NTU156	27.92 *	37.50 *	1.34		[21]
Chloroquine	FIPV-II 79-1146	0.38	82.31 ***	216		[22]
Hydroxychloroquine	FIPV-I KU-2	48.7 *	515.7 (timing not specified)	10.59		[20]
	FIPV-II 79-1146	30.3 *	515.7 (timing not specified)	17.02		[20]
GS-441524	FIPV-II NTU156	3.5 *	>100 *	>28.57	clinical trials [23,24]	[21]
	FIPV-II 79-1146	0.78 *	>100–>10 **			[25]
Mefloquine	FIPV-II 79-1146	0.74–5.71 *–7.89 **	15.13 ***		Pharmacokinetics only [26]	[22]
Ribavirin	FIPV-II 79-1146	41.7 **	NA	NA	SPF cats [27]	[28]
Adenosine arabinoside	FIPV-II 79-1146	125	NA	NA		[28]
3-Deazaguanosine	FIPV-II 79-1146	10.37 **	323.32 **	31.18		[29]
6-azauridine	FIPV-II 79-1146	0.16 **	19.4 **	121.25		[29]
siRNA L2 (leader 2)	FIPV-II 79-1146	0.0012 ¤	NA	NA		[30]
siRNA N1 (nucleocapsid 1)	FIPV-II 79-1146	0.0018 ¤	NA	NA		[30]
Fusidic acid	FIPV-II 79-1146	75 **	232.56 **	3,1		[29]
Hygromycin B	FIPV-II 79-1146	2.25 **	29.94 **	13,31		[29]
GC376	FIPV-II 79-1146	0.04	>150	>3750	Clinical trials [31,32]	[33]
	FIPV-II NTU156	0.4–2 *	>50 *	>25		[34]
NPI64	FIPV-II 79-1146	0.04	61.91	1547.75	Pharmacokinetics only [31]	[33]
Nelfinavir	FIPV-IINTU156	8.19 °	11.45 **			[35]
U18666A	FIPV II 79-1146	>50 **	97.6 **	488	SPF cats [36]	[36]
	FIPV-I KU-2	0.2 **				
Itraconazol	FIPV-I KU-2	0.219 **	208 **	950.6	SPF cats [37]	[37]
	FIPV-I UCD1	0.597 **	208 **	348.7		
	FIPV-I UCD4	0.146 **	208 **	1425.9		
	FIPV-II 79-1146	>160 **	208 **	<1.3		
	FIPV-I KU-2	0.7	121 **	172.9		[38]

^1^ indicates the timing post-infection: * 48 h, ** 72 h, *** 96 h, ¤ 36 h, ° 15 h, °° for 6 h and no symbol 24 h. SI is the ratio of CC50/IC50. GNA: *Galanthus nivalis* agglutinin. UDA: *Urtica dioica* agglutinin. HHA: *Hippeastrum* hybrid agglutinin. NA: Not Available, SPF: Specific Pathogens Free, SI: Selectivity Index.

**Table 2:** FIPV antivirals effect on other coronaviruses.

Inhibitor	Antiviral Effect on Other Coronaviruses
Dyphillin	SARS-CoV-2 [84]
GNA	IBV, TGEV [18], SARS-CoV [85], MHV [86]
HHA	IBV, TGEV [18], SARS-CoV [85], MHV [86]
UDA	IBV, TGEV [18], SARS-CoV [85], MHV [86]
Chloroquine	BCoV [87], SARS-CoV [88], HCoV-229E [44], HCoV-OC43 [89], MERS-CoV [90], PHEV [91], SARS-CoV-2 [92]
Hydroxychloroquine	SARS-CoV-2 [93]
Mefloquine	SARS-CoV-2 [94]
Ribavirin	MHV [95], SARS-CoV [96]
6-azauridine	MHV [95], SARS-CoV [96]
GS-441524	MHV, SARS-CoV, MERS-CoV [97], SARS-CoV-2 [98]
Hygromycin B	MHV [99], BCoV [100]
GC376	SARS-CoV-2 [81], TGEV, SARS-CoV, MHV, HCoV-229E, BCoV [62], HCoV-OC43, HCoV-NL63, MERS-CoV [101]
U18666A	SARS-CoV, MERS-CoV, HCoV-NL63, HCoV-229E [102]
Itraconazol	SARS-CoV-2 [66] MHV [95]

SARS: Severe acute respiratory syndrome, TGEV: Transmissible gastroenteritis virus, MHV: Murine hepatitis virus, MERS: Middle-East respiratory syndrome, BCoV: Bovine coronavirus, PHEV: Porcine Hemagglutinating Encephalomyelitis Virus, IBV: Infectious bronchitis virus GNA: *Galanthus nivalis* agglutinin. UDA: *Urtica dioica* agglutinin. HHA: *Hippeastrum hybrid* agglutinin.

## References

[1] Ye G., Wang X., Tong X., Shi Y., Fu Z.F., Peng G. (2020). Structural Basis for Inhibiting Porcine Epidemic Diarrhea Virus Replication with the 3C-Like Protease Inhibitor GC376. Viruses.

[2] Prince T., Smith S.L., Radford A.D., Solomon T., Hughes G.L., Patterson E.I. (2021). SARS-CoV-2 Infections in Animals: Reservoirs for Reverse Zoonosis and Models for Study. Viruses.

[3] Mackay I.M., Arden K.E. (2015). MERS Coronavirus: Diagnostics, Epidemiology and Transmission. Virol. J..

[4] Schalk A.F. (1931). An Apparently New Respiratory Disease of Baby Chicks. J. Am. Vet. Med. Assoc..

[5] Hamre D., Procknow J.J. (1966). A New Virus Isolated from the Human Respiratory Tract. Proc. Soc. Exp. Biol. Med..

[6] Cavanagh D. (2007). Coronavirus Avian Infectious Bronchitis Virus. Vet. Res..

[7] Haake C., Cook S., Pusterla N., Murphy B. (2020). Coronavirus Infections in Companion Animals: Virology, Epidemiology, Clinical and Pathologic Features. Viruses.

[8] Amer H.M. (2018). Bovine-like Coronaviruses in Domestic and Wild Ruminants. Anim. Health Res. Rev..

[9] Williams B.H., Kiupel M., West K.H., Raymond J.T., Grant C.K., Glickman L.T. (2000). Coronavirus-Associated Epizootic Catarrhal Enteritis in Ferrets. J. Am. Vet. Med. Assoc..

[10] Pedersen N.C. (2014). An Update on Feline Infectious Peritonitis: Diagnostics and Therapeutics. Vet. J..

[11] Leibowitz J., Kaufman G., Liu P. (2011). Coronaviruses: Propagation, Quantification, Storage, and Construction of Recombinant Mouse Hepatitis Virus. Curr. Protoc. Microbiol..

[12] Rottier P.J.M., Nakamura K., Schellen P., Volders H., Haijema B.J. (2005). Acquisition of Macrophage Tropism during the Pathogenesis of Feline Infectious Peritonitis Is Determined by Mutations in the Feline Coronavirus Spike Protein. J. Virol..

[13] Kummrow M., Meli M.L., Haessig M., Goenczi E., Poland A., Pedersen N.C., Hofmann-Lehmann R., Lutz H. (2005). Feline Coronavirus Serotypes 1 and 2: Seroprevalence and Association with Disease in Switzerland. Clin. Vaccine Immunol..

[14] Bálint Á., Farsang A., Szeredi L., Zádori Z., Belák S. (2014). Recombinant Feline Coronaviruses as Vaccine Candidates Confer Protection in SPF but Not in Conventional Cats. Vet. Microbiol..

[15] Fehr D., Holznagel E., Bolla S., Hauser B., Herrewegh A.A., Horzinek M.C., Lutz H. (1997). Placebo-Controlled Evaluation of a Modified Life Virus Vaccine against Feline Infectious Peritonitis: Safety and Efficacy under Field Conditions. Vaccine.

[16] Liu I.-J., Tsai W.-T., Hsieh L.-E., Chueh L.-L. (2013). Peptides Corresponding to the Predicted Heptad Repeat 2 Domain of the Feline Coronavirus Spike Protein Are Potent Inhibitors of Viral Infection. PLoS ONE.

[17] Hu C.-M.J., Chang W.-S., Fang Z.-S., Chen Y.-T., Wang W.-L., Tsai H.-H., Chueh L.-L., Takano T., Hohdatsu T., Chen H.-W. (2017). Nanoparticulate Vacuolar ATPase Blocker Exhibits Potent Host-Targeted Antiviral Activity against Feline Coronavirus. Sci. Rep..

[18] Van der Meer F.J.U.M., de Haan C.A.M., Schuurman N.M.P., Haijema B.J., Peumans W.J., Van Damme E.J.M., Delputte P.L., Balzarini J., Egberink H.F. (2007). Antiviral Activity of Carbohydrate-Binding Agents against Nidovirales in Cell Culture. Antivir. Res..

[19] Takano T., Katoh Y., Doki T., Hohdatsu T. (2013). Effect of Chloroquine on Feline Infectious Peritonitis Virus Infection in Vitro and in Vivo. Antivir. Res..

[20] Takano T., Satoh K., Doki T., Tanabe T., Hohdatsu T. (2020). Antiviral Effects of Hydroxychloroquine and Type I Interferon on In Vitro Fatal Feline Coronavirus Infection. Viruses.

[21] Yang C.-W., Peng T.-T., Hsu H.-Y., Lee Y.-Z., Wu S.-H., Lin W.-H., Ke Y.-Y., Hsu T.-A., Yeh T.-K., Huang W.-Z. (2020). Repurposing Old Drugs as Antiviral Agents for Coronaviruses. Biomed. J..

[22] McDonagh P., Sheehy P.A., Norris J.M. (2014). Identification and Characterisation of Small Molecule Inhibitors of Feline Coronavirus Replication. Vet. Microbiol..

[23] Pedersen N.C., Perron M., Bannasch M., Montgomery E., Murakami E., Liepnieks M., Liu H. (2019). Efficacy and Safety of the Nucleoside Analog GS-441524 for Treatment of Cats with Naturally Occurring Feline Infectious Peritonitis. J. Feline Med. Surg..

[24] Dickinson P.J., Bannasch M., Thomasy S.M., Murthy V.D., Vernau K.M., Liepnieks M., Montgomery E., Knickelbein K.E., Murphy B., Pedersen N.C. (2020). Antiviral Treatment Using the Adenosine Nucleoside Analogue GS-441524 in Cats with Clinically Diagnosed Neurological Feline Infectious Peritonitis. J. Vet. Intern. Med..

[25] Murphy B.G., Perron M., Murakami E., Bauer K., Park Y., Eckstrand C., Liepnieks M., Pedersen N.C. (2018). The Nucleoside Analog GS-441524 Strongly Inhibits Feline Infectious Peritonitis (FIP) Virus in Tissue Culture and Experimental Cat Infection Studies. Vet. Microbiol..

[26] Yu J., Kimble B., Norris J.M., Govendir M. (2020). Pharmacokinetic Profile of Oral Administration of Mefloquine to Clinically Normal Cats: A Preliminary In-Vivo Study of a Potential Treatment for Feline Infectious Peritonitis (FIP). Animals.

[27] Weiss R.C., Oostrom-Ram T. (1989). Inhibitory Effects of Ribavirin Alone or Combined with Human Alpha Interferon on Feline Infectious Peritonitis Virus Replication in Vitro. Vet. Microbiol..

[28] Barlough J.E., Scott F.W. (1990). Effectiveness of Three Antiviral Agents against FIP Virus in Vitro. Vet. Rec..

[29] Barlough J.E., Shacklett B.L. (1994). Antiviral Studies of Feline Infectious Peritonitis Virus in Vitro. Vet. Rec..

[30] McDonagh P., Sheehy P.A., Norris J.M. (2015). Combination SiRNA Therapy against Feline Coronavirus Can Delay the Emergence of Antiviral Resistance in Vitro. Vet. Microbiol..

[31] Kim Y., Liu H., Galasiti Kankanamalage A.C., Weerasekara S., Hua D.H., Groutas W.C., Chang K.-O., Pedersen N.C. (2016). Reversal of the Progression of Fatal Coronavirus Infection in Cats by a Broad-Spectrum Coronavirus Protease Inhibitor. PLoS Pathog..

[32] Pedersen N.C., Kim Y., Liu H., Galasiti Kankanamalage A.C., Eckstrand C., Groutas W.C., Bannasch M., Meadows J.M., Chang K.-O. (2018). Efficacy of a 3C-like Protease Inhibitor in Treating Various Forms of Acquired Feline Infectious Peritonitis. J. Feline Med. Surg..

[33] Kim Y., Mandadapu S.R., Groutas W.C., Chang K.-O. (2013). Potent Inhibition of Feline Coronaviruses with Peptidyl Compounds Targeting Coronavirus 3C-like Protease. Antivir. Res..

[34] Ke Y.-Y., Peng T.-T., Yeh T.-K., Huang W.-Z., Chang S.-E., Wu S.-H., Hung H.-C., Hsu T.-A., Lee S.-J., Song J.-S. (2020). Artificial Intelligence Approach Fighting COVID-19 with Repurposing Drugs. Biomed. J..

[35] Hsieh L.-E., Lin C.-N., Su B.-L., Jan T.-R., Chen C.-M., Wang C.-H., Lin D.-S., Lin C.-T., Chueh L.-L. (2010). Synergistic Antiviral Effect of Galanthus Nivalis Agglutinin and Nelfinavir against Feline Coronavirus. Antivir. Res..

[36] Doki T., Tarusawa T., Hohdatsu T., Takano T. (2020). In Vivo Antiviral Effects of U18666A Against Type I Feline Infectious Peritonitis Virus. Pathogens.

[37] Takano T., Akiyama M., Doki T., Hohdatsu T. (2019). Antiviral Activity of Itraconazole against Type I Feline Coronavirus Infection. Vet. Res..

[38] Doki T., Toda M., Hasegawa N., Hohdatsu T., Takano T. (2020). Therapeutic Effect of an Anti-Human-TNF-Alpha Antibody and Itraconazole on Feline Infectious Peritonitis. Arch. Virol..

[39] Regan A.D., Whittaker G.R. (2008). Utilization of DC-SIGN for Entry of Feline Coronaviruses into Host Cells. J. Virol..

[40] Shirato K., Kanou K., Kawase M., Matsuyama S. (2017). Clinical Isolates of Human Coronavirus 229E Bypass the Endosome for Cell Entry. J. Virol..

[41] Van Hamme E., Dewerchin H.L., Cornelissen E., Verhasselt B., Nauwynck H.J. (2008). Clathrin- and Caveolae-Independent Entry of Feline Infectious Peritonitis Virus in Monocytes Depends on Dynamin. J. Gen. Virol..

[42] Doki T., Takano T., Koyama Y., Hohdatsu T. (2015). Identification of the Peptide Derived from S1 Domain That Inhibits Type I and Type II Feline Infectious Peritonitis Virus Infection. Virus Res..

[43] Chen H.-W., Cheng J.X., Liu M.-T., King K., Peng J.-Y., Zhang X.-Q., Wang C.-H., Shresta S., Schooley R.T., Liu Y.-T. (2013). Inhibitory and Combinatorial Effect of Diphyllin, a v-ATPase Blocker, on Influenza Viruses. Antivir. Res..

[44] Kono M., Tatsumi K., Imai A.M., Saito K., Kuriyama T., Shirasawa H. (2008). Inhibition of Human Coronavirus 229E Infection in Human Epithelial Lung Cells (L132) by Chloroquine: Involvement of P38 MAPK and ERK. Antivir. Res..

[45] Murray S.M., Down C.M., Boulware D.R., Stauffer W.M., Cavert W.P., Schacker T.W., Brenchley J.M., Douek D.C. (2010). Reduction of Immune Activation with Chloroquine Therapy during Chronic HIV Infection. J. Virol..

[46] Savarino A., Boelaert J.R., Cassone A., Majori G., Cauda R. (2003). Effects of Chloroquine on Viral Infections: An Old Drug against Today’s Diseases?. Lancet Infect. Dis..

[47] Vincent M.J., Bergeron E., Benjannet S., Erickson B.R., Rollin P.E., Ksiazek T.G., Seidah N.G., Nichol S.T. (2005). Chloroquine Is a Potent Inhibitor of SARS Coronavirus Infection and Spread. Virol. J..

[48] Liu J., Cao R., Xu M., Wang X., Zhang H., Hu H., Li Y., Hu Z., Zhong W., Wang M. (2020). Hydroxychloroquine, a Less Toxic Derivative of Chloroquine, Is Effective in Inhibiting SARS-CoV-2 Infection in Vitro. Cell Discov..

[49] McChesney E.W. (1983). Animal Toxicity and Pharmacokinetics of Hydroxychloroquine Sulfate. Am. J. Med..

[50] Sawicki S.G., Sawicki D.L. (1995). Coronaviruses Use Discontinuous Extension for Synthesis of Subgenome-Length Negative Strands. Adv. Exp. Med. Biol..

[51] Yin Y., Li T., Wang C., Liu X., Ouyang H., Ji W., Liu J., Liao X., Li J., Hu C. (2021). A Retrospective Study of Clinical and Laboratory Features and Treatment on Cats Highly Suspected of Feline Infectious Peritonitis in Wuhan, China. Sci. Rep..

[52] Addie D.D., Curran S., Bellini F., Crowe B., Sheehan E., Ukrainchuk L., Decaro N. (2020). Oral Mutian®X Stopped Faecal Feline Coronavirus Shedding by Naturally Infected Cats. Res. Vet. Sci..

[53] Addie D.D., Covell-Ritchie J., Jarrett O., Fosbery M. (2020). Rapid Resolution of Non-Effusive Feline Infectious Peritonitis Uveitis with an Oral Adenosine Nucleoside Analogue and Feline Interferon Omega. Viruses.

[54] Jones S., Novicoff W., Nadeau J., Evans S. (2021). Unlicensed GS-441524-Like Antiviral Therapy Can Be Effective for at-Home Treatment of Feline Infectious Peritonitis. Animals.

[55] Weiss R.C., Cox N.R., Martinez M.L. (1993). Evaluation of Free or Liposome-Encapsulated Ribavirin for Antiviral Therapy of Experimentally Induced Feline Infectious Peritonitis. Res. Vet. Sci..

[56] Merigan T.C. (1982). Systemic Antivirals for Therapy of Herpesvirus Diseases. Dev. Biol. Stand..

[57] Qureshi A., Tantray V.G., Kirmani A.R., Ahangar A.G. (2018). A Review on Current Status of Antiviral siRNA. Rev. Med. Virol..

[58] McDonagh P., Sheehy P.A., Norris J.M. (2011). In Vitro Inhibition of Feline Coronavirus Replication by Small Interfering RNAs. Vet. Microbiol..

[59] Knauert M.P., Glazer P.M. (2001). Triplex Forming Oligonucleotides: Sequence-Specific Tools for Gene Targeting. Hum. Mol. Genet..

[60] Choong O.K., Mehrbod P., Tejo B.A., Omar A.R. (2014). In Vitro Antiviral Activity of Circular Triple Helix Forming Oligonucleotide RNA towards Feline Infectious Peritonitis Virus Replication. BioMed Res. Int..

[61] Kim Y., Shivanna V., Narayanan S., Prior A.M., Weerasekara S., Hua D.H., Kankanamalage A.C.G., Groutas W.C., Chang K.-O. (2015). Broad-Spectrum Inhibitors against 3C-Like Proteases of Feline Coronaviruses and Feline Caliciviruses. J. Virol..

[62] Kim Y., Lovell S., Tiew K.-C., Mandadapu S.R., Alliston K.R., Battaile K.P., Groutas W.C., Chang K.-O. (2012). Broad-Spectrum Antivirals against 3C or 3C-Like Proteases of Picornaviruses, Noroviruses, and Coronaviruses. J. Virol..

[63] Perera K.D., Rathnayake A.D., Liu H., Pedersen N.C., Groutas W.C., Chang K.-O., Kim Y. (2019). Characterization of Amino Acid Substitutions in Feline Coronavirus 3C-like Protease from a Cat with Feline Infectious Peritonitis Treated with a Protease Inhibitor. Vet. Microbiol..

[64] Yamamoto N., Yang R., Yoshinaka Y., Amari S., Nakano T., Cinatl J., Rabenau H., Doerr H.W., Hunsmann G., Otaka A. (2004). HIV Protease Inhibitor Nelfinavir Inhibits Replication of SARS-Associated Coronavirus. Biochem. Biophys. Res. Commun..

[65] Takano T., Endoh M., Fukatsu H., Sakurada H., Doki T., Hohdatsu T. (2017). The Cholesterol Transport Inhibitor U18666A Inhibits Type I Feline Coronavirus Infection. Antivir. Res..

[66] Van Damme E., De Meyer S., Bojkova D., Ciesek S., Cinatl J., De Jonghe S., Jochmans D., Leyssen P., Buyck C., Neyts J. (2021). In Vitro Activity of Itraconazole against SARS-CoV-2. J. Med. Virol..

[67] Boothe D.M., Herring I., Calvin J., Way N., Dvorak J. (1997). Itraconazole Disposition after Single Oral and Intravenous and Multiple Oral Dosing in Healthy Cats. Am. J. Vet. Res..

[68] Kameshima S., Kimura Y., Doki T., Takano T., Park C.-H., Itoh N. (2020). Clinical Efficacy of Combination Therapy of Itraconazole and Prednisolone for Treating Effusive Feline Infectious Peritonitis. J. Vet. Med. Sci..

[69] Tanaka Y., Sato Y., Osawa S., Inoue M., Tanaka S., Sasaki T. (2012). Suppression of Feline Coronavirus Replication In Vitro by Cyclosporin A. Vet. Res..

[70] Tanaka Y., Sato Y., Takahashi D., Matsumoto H., Sasaki T. (2015). Treatment of a Case of Feline Infectious Peritonitis with Cyclosporin A. Vet. Rec. Case Rep..

[71] Mochizuki M., Nakatani H., Yoshida M. (1994). Inhibitory Effects of Recombinant Feline Interferon on the Replication of Feline Enteropathogenic Viruses in Vitro. Vet. Microbiol..

[72] Ritz S., Egberink H., Hartmann K. (2007). Effect of Feline Interferon-Omega on the Survival Time and Quality of Life of Cats with Feline Infectious Peritonitis. J. Vet. Intern. Med..

[73] Weiss R.C., Toivio-Kinnucan M. (1988). Inhibition of Feline Infectious Peritonitis Virus Replication by Recombinant Human Leukocyte (Alpha) Interferon and Feline Fibroblastic (Beta) Interferon. Am. J. Vet. Res..

[74] Weiss R.C., Cox N.R., Oostrom-Ram T. (1990). Effect of Interferon or Propionibacterium Acnes on the Course of Experimentally Induced Feline Infectious Peritonitis in Specific-Pathogen-Free and Random-Source Cats. Am. J. Vet. Res..

[75] Addie D., Belák S., Boucraut-Baralon C., Egberink H., Frymus T., Gruffydd-Jones T., Hartmann K., Hosie M.J., Lloret A., Lutz H. (2009). Feline Infectious Peritonitis. ABCD Guidelines on Prevention and Management. J. Feline Med. Surg..

[76] Legendre A.M., Kuritz T., Galyon G., Baylor V.M., Heidel R.E. (2017). Polyprenyl Immunostimulant Treatment of Cats with Presumptive Non-Effusive Feline Infectious Peritonitis In a Field Study. Front. Vet. Sci..

[77] González-Grande R., Jiménez-Pérez M., González Arjona C., Mostazo Torres J. (2016). New Approaches in the Treatment of Hepatitis C. World J. Gastroenterol..

[78] Heo Y.-A., Deeks E.D. (2018). Sofosbuvir/Velpatasvir/Voxilaprevir: A Review in Chronic Hepatitis C. Drugs.

[79] Perelson A.S., Essunger P., Cao Y., Vesanen M., Hurley A., Saksela K., Markowitz M., Ho D.D. (1997). Decay Characteristics of HIV-1-Infected Compartments during Combination Therapy. Nature.

[80] Cook S.E., Vogel H., Castillo D., Olsen M., Pedersen N., Murphy B.G. (2020). A Rational Approach to Identifying Effective Combined Anticoronaviral Therapies against Feline Coronavirus. bioRxiv.

[81] Vuong W., Fischer C., Khan M.B., van Belkum M.J., Lamer T., Willoughby K.D., Lu J., Arutyunova E., Joyce M.A., Saffran H.A. (2021). Improved SARS-CoV-2 Mpro Inhibitors Based on Feline Antiviral Drug GC376: Structural Enhancements, Increased Solubility, and Micellar Studies. Eur. J. Med. Chem..

[82] Yan V.C., Muller F.L. (2020). Advantages of the Parent Nucleoside GS-441524 over Remdesivir for COVID-19 Treatment. ACS Med. Chem. Lett..

[83] Barre A., Van Damme E.J.M., Simplicien M., Le Poder S., Klonjkowski B., Benoist H., Peyrade D., Rougé P. (2021). Man-Specific Lectins from Plants, Fungi, Algae and Cyanobacteria, as Potential Blockers for SARS-CoV, MERS-CoV and SARS-CoV-2 (COVID-19) Coronaviruses: Biomedical Perspectives. Cells.

[84] Stefanik M., Strakova P., Haviernik J., Miller A.D., Ruzek D., Eyer L. (2021). Antiviral Activity of Vacuolar ATPase Blocker Diphyllin against SARS-CoV-2. Microorganisms.

[85] Keyaerts E., Vijgen L., Pannecouque C., Van Damme E., Peumans W., Egberink H., Balzarini J., Van Ranst M. (2007). Plant Lectins Are Potent Inhibitors of Coronaviruses by Interfering with Two Targets in the Viral Replication Cycle. Antivir. Res..

[86] Van der Meer F.J.U.M., de Haan C.A.M., Schuurman N.M.P., Haijema B.J., Verheije M.H., Bosch B.J., Balzarini J., Egberink H.F. (2007). The Carbohydrate-Binding Plant Lectins and the Non-Peptidic Antibiotic Pradimicin A Target the Glycans of the Coronavirus Envelope Glycoproteins. J. Antimicrob. Chemother..

[87] Payne H.R., Storz J., Henk W.G. (1990). Initial Events in Bovine Coronavirus Infection: Analysis through Immunogold Probes and Lysosomotropic Inhibitors. Arch. Virol..

[88] Keyaerts E., Vijgen L., Maes P., Neyts J., Van Ranst M. (2004). In Vitro Inhibition of Severe Acute Respiratory Syndrome Coronavirus by Chloroquine. Biochem. Biophys. Res. Commun..

[89] Keyaerts E., Li S., Vijgen L., Rysman E., Verbeeck J., Van Ranst M., Maes P. (2009). Antiviral Activity of Chloroquine against Human Coronavirus OC43 Infection in Newborn Mice. Antimicrob. Agents Chemother..

[90] De Wilde A.H., Jochmans D., Posthuma C.C., Zevenhoven-Dobbe J.C., van Nieuwkoop S., Bestebroer T.M., van den Hoogen B.G., Neyts J., Snijder E.J. (2014). Screening of an FDA-Approved Compound Library Identifies Four Small-Molecule Inhibitors of Middle East Respiratory Syndrome Coronavirus Replication in Cell Culture. Antimicrob. Agents Chemother..

[91] Ding N., Zhao K., Lan Y., Li Z., Lv X., Su J., Lu H., Gao F., He W. (2017). Induction of Atypical Autophagy by Porcine Hemagglutinating Encephalomyelitis Virus Contributes to Viral Replication. Front. Cell. Infect. Microbiol..

[92] Wang M., Cao R., Zhang L., Yang X., Liu J., Xu M., Shi Z., Hu Z., Zhong W., Xiao G. (2020). Remdesivir and Chloroquine Effectively Inhibit the Recently Emerged Novel Coronavirus (2019-NCoV) in Vitro. Cell Res..

[93] Yao X., Ye F., Zhang M., Cui C., Huang B., Niu P., Liu X., Zhao L., Dong E., Song C. (2020). In Vitro Antiviral Activity and Projection of Optimized Dosing Design of Hydroxychloroquine for the Treatment of Severe Acute Respiratory Syndrome Coronavirus 2 (SARS-CoV-2). Clin. Infect. Dis..

[94] Shionoya K., Yamasaki M., Iwanami S., Ito Y., Fukushi S., Ohashi H., Saso W., Tanaka T., Aoki S., Kuramochi K. (2021). Mefloquine, a Potent Anti-Severe Acute Respiratory Syndrome-Related Coronavirus 2 (SARS-CoV-2) Drug as an Entry Inhibitor in Vitro. Front. Microbiol..

[95] Cao J., Forrest J.C., Zhang X. (2015). A Screen of the NIH Clinical Collection Small Molecule Library Identifies Potential Anti-Coronavirus Drugs. Antivir. Res..

[96] Cinatl J., Morgenstern B., Bauer G., Chandra P., Rabenau H., Doerr H. (2003). Glycyrrhizin, an Active Component of Liquorice Roots, and Replication of SARS-Associated Coronavirus. Lancet.

[97] Agostini M.L., Andres E.L., Sims A.C., Graham R.L., Sheahan T.P., Lu X., Smith E.C., Case J.B., Feng J.Y., Jordan R. (2018). Coronavirus Susceptibility to the Antiviral Remdesivir (GS-5734) Is Mediated by the Viral Polymerase and the Proofreading Exoribonuclease. MBio.

[98] Li Y., Cao L., Li G., Cong F., Li Y., Sun J., Luo Y., Chen G., Li G., Wang P. (2021). Remdesivir Metabolite GS-441524 Effectively Inhibits SARS-CoV-2 Infection in Mouse Models. J. Med. Chem..

[99] Macintyre G., Curry B., Wong F., Anderson R. (1991). Hygromycin B Therapy of a Murine Coronaviral Hepatitis. Antimicrob. Agents Chemother..

[100] Kapil S., Richardson K.L., Maag T.R., Goyal S.M. (1999). Characterization of Bovine Coronavirus Isolates/from Eight Different States in the USA. Vet. Microbiol..

[101] Hu Y., Ma C., Szeto T., Hurst B., Tarbet B., Wang J. (2020). Boceprevir, Calpain Inhibitors II and XII, and GC-376 Have Broad-Spectrum Antiviral Activity against Coronaviruses in Cell Culture. bioRxiv.

[102] Wrensch F., Winkler M., Pöhlmann S. (2014). IFITM Proteins Inhibit Entry Driven by the MERS-Coronavirus Spike Protein: Evidence for Cholesterol-Independent Mechanisms. Viruses.

